# Structural Damage Identification of Bridges from Passing Test Vehicles

**DOI:** 10.3390/s18114035

**Published:** 2018-11-19

**Authors:** Yang Yang, Yuanhao Zhu, Li Lei Wang, Bao Yulong Jia, Ruoyu Jin

**Affiliations:** 1 MOE Key Laboratory of New Technology for Construction of Cities in Mountain Area, and School of Civil Engineering, Chongqing University, Chongqing 400045, China; m18723232480@163.com (Y.Z.); leeleiwang@163.com (L.L.W.); 2 Horoy Property Group (Shenzhen) Co., Ltd., Shenzhen 518000, China; aijiangmini@163.com; 3 Subject of Built Environment, School of Environment and Technology, University of Brighton, Brighton BN2 4GJ, UK; R.Jin@brighton.ac.uk

**Keywords:** bending stiffness, damage identification, environmental noise, bridge, test vehicle

## Abstract

This paper presents two approaches for the structural damage identification of a bridge from the dynamic response recorded from a test vehicle during its passage over the bridge. Using the acceleration response recorded by the vibration sensors mounted on a test vehicle during its passage over the bridge, along with the computed displacement response, the bending stiffness of the bridge can be determined using either: (1) the frequency-domain method based on the improved directed stiffness method with the identified frequency and corresponding mode shape, or (2) the time-domain method based on the residual vector of the least squares method with a fourth-order displacement moment. By comparing the bending stiffness values identified from the vehicle-collected data for the bridge under the undamaged and damaged states that are monitored regularly by the test vehicle, the bridge damage location and severity can be identified. Through numerical simulations and field tests, the present approaches are shown to be effective and feasible.

## 1. Introduction

The physical properties of a structure such as stiffness and mass are important for structural health monitoring, because variations in these properties indicate the direct occurrence of damage. In most of the damage detection schemes, the mass of a structure is usually assumed to remain unchanged before and after the occurrence of damage. Accordingly, the change in stiffness of a structure is the most crucial dynamic property for damage identification.

Hou et al. [1] presented comprehensive reviews for the literature on the damage detection of structures. Amezquita-Sanchez and Adeli [2] presented a state-of-the-art review of recent articles on signal processing techniques for vibration-based SHM. Considering the bending stiffness index identification, Maeck [3,4] proposed the bending stiffness estimation approach for structures using the frequencies, mode shapes, and their derivatives, which is called the direct stiffness calculation (DSC) technique. Xu et al. [5,6] proposed the method of statistical moment-based damage detection (SMBDD) for inversely calculating the stiffness of steel-framed structures, which is sensitive to local structural damage, but insensitive to measurement noise. By integrating the generalized pattern search algorithm with the indirect identification technique using a passing vehicle, Li et al. [7] calculated the bending stiffness of a bridge, and pointed out that parameters such as the penalty values and mesh features should be further studied. Considering the difficulty of choosing the appropriate penalty factors for use in the DSC technique, Yang et al. [8,9,10] calculated the stiffness through an improved DSC technique for application to practical structures, which was verified both theoretically and experimentally.

Most of the identification techniques for bridges are referred to as the direct identification method, since they rely on the data collected by the vibration sensors that are directly mounted on the bridge. Blachowski et al. [11] proposed the axial strain accelerations degree of dispersion method with PCB piezoelectric accelerometers arranged directly in a truss structure. Kim et al. [12] studied Nair’s damage indicator and its statistical pattern with a field experiment of a real continuous steel Gerber-truss bridge by the acceleration response of the bridge. Sevillano et al. [13] used a modal interval analysis method to address the uncertainty in vibration-based damage detection of a concrete frame. Yang et al. [14] proposed the deterministic and stochastic approaches for damage identification of experimental benchmark Reinforced Concrete (RC) frame model based on the fusing damage index by combining two types of statistical moment. Mao and Wang et al. [15,16] investigated the relationships between the dynamic properties and the environmental factors, especially the temperature based on the one-year monitoring data under normal operating conditions and one typhoon monitoring data by the sensors directly arranged on a Sutong Cable-Stayed Bridge. The indirect identification technique differs from the conventional direct method for measuring the bridge dynamic properties in that no vibration sensors need to be installed on the bridge. Rather, only one or a few vibration sensors need to be mounted on an instrumented test vehicle to record its response when passing over the bridge, from which the dynamic properties of the bridge are identified. The indirect identification technique, using a test vehicle to extract the first few bridge frequencies, was first proposed in 2004 by Yang et al. [17,18], and subsequently validated experimentally by Yang and Lin [19]. Originally, the main focus of the indirect identification technique is to extract the frequencies of the bridge, which is the most basic parameter related to the health status of a bridge. This technique is based on the transformation of the recorded data for the test vehicle from the time domain to the frequency domain using fast Fourier transform (FFT) [18,19], empirical mode decomposition [20], or other techniques [21,22,23,24]. Along these lines, Feng and Feng [21] proposed a bridge damage detection procedure that utilizes the vehicle-induced displacement response of the bridge, particularly, the curvature of the first mode shape, for simulated damage cases. OBrien and Keenahan [22] used a vehicle equipped with traffic speed deflectometers (TSDs) for determining the apparent profile of a bridge by an optimization algorithm, and showed that the time-shifted difference in the apparent profile can be probably used as a damage indicator of the bridge in the presence of noise by simulation. Behroozinia and Khaleghian et al. [23] presented a finite element model of the intelligent tire by using implicit dynamic analysis for defect tire detection. McGetrick et al. [24] used the test vehicle to identify the frequency and damping of a bridge, considering both smooth and rough bridge surfaces, and various vehicle speeds. It is noted that the application of the indirect method has been mainly focused on the frequency, damping, and indirect parameters with relation to the damage of the bridge in previous studies. More significantly, other properties of the bridge—particularly those for directly identifying stiffness, which reveals that the health status of a bridge—have not been evaluated using the indirect technique.

In this paper, it is assumed that the test vehicle is allowed to regularly monitor the bridge termly. The response of the test vehicle recorded during the *current* travel is assumed to be the *damaged* state and that of the *previous* travel is assumed to be the *undamaged* state. If no damage is detected by comparison of the two states, the current state is reset as the undamaged one, and another monitoring continues. By comparing the bending stiffness values identified from the vehicle-collected data for the bridge under the undamaged and damaged states monitored regularly by the test vehicle, the bridge damage location and severity can be identified based on the undamaged state. Only the *acceleration response* of the test vehicle is measured, and the *displacement response* is calculated by integration. Compared with previous studies, the bending stiffness estimation approach for each element of the bridge for damage identification is the main object of this paper, and the more prominent advantage of the indirect technique. The technique was developed by Yang et al. [25], and is used to obtain the mode shape of the monitored bridge by the test vehicle response. This mode shape is subsequently utilized to calculate the bending stiffness of the bridge, which is referred to as the *frequency domain method*. Using this method, a reliance on assumed penalty factors is necessary. On the other hand, making use of the relationship between the displacement response of the test vehicle and the bending stiffness of the bridge [17], the fourth-order statistical moment (the fourth-order statistical moment of structural response is expressed in terms of a probability density function (PDF) p(x) as $M_{4}=\int_{-\infty }^{+\infty }{\left(x-\bar{x}\right)}^{4}p\left(x\right)dx$, where x is the structural response with $\bar{x}$ as its mean value.) of the displacement response of the bridge is computed using the procedure documented in Xu et al. [5,6]. Subsequently, the bending stiffness of the bridge is acquired for damage detection, which is referred to as the *time-domain method*.

The adopted frequency domain method is a fast, initial evaluation technique for detecting the structural condition, since no optimization is required. In contrast, the adopted time domain method is a time-consuming, meticulous evaluation technique for damage detection, since all of the relevant parameters have to be optimized. In this paper, the used response data of the test vehicle are generated by simulation and field experimental tests, where the paper is focused on the *feasibility* of the indirect approach for damage detection, making use of such simulated and recorded data, which can be used for updating a real-time identification of structural damage in a timely manner.

## 2. Theoretical Background and Formulations

### 2.1. Frequency Domain Method

**Figure 1:**
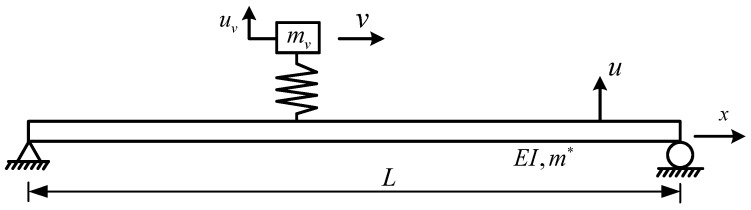
Moving test vehicle over a bridge.

Figure 1 shows the mathematical model for a test vehicle moving on a bridge. In this model, the vehicle is simplified as a moving mass *m_v_*, supported by a spring of stiffness *k_v_*; the bridge is a simply-supported beam of span *L*, uniform mass density m* per unit length, and uniform bending rigidity *EI*. To focus on the physical behavior of the vehicle, the following assumptions are adopted without a loss of generality for the problem. (1) Road surface roughness is ignored in the derivation, but is included in one of the studied numerical cases and field tests to evaluate the influence of this assumption. (2) Vehicle mass is negligibly small in comparison with the bridge mass. (3) Prior to the arrival of the test vehicle, the bridge remains at rest, i.e., zero initial conditions are assumed for the bridge, which is acceptable because the bridge vibrations caused by ambient excitations are small compared to those caused by moving vehicular loads. (4) Damping is neglected for both the vehicle and the bridge, which is acceptable, because the vibrations of both the vehicle and the bridge under moving loads are forced vibrations where damping is usually insignificant. (5) The test vehicle travels at a constant speed, *v*, during its passage over the bridge.

The equations of motion can be written for the vehicle and bridge as follows:

(1) $$m_{v}{\ddot{u}}_{v}(t)+k_{v}(u_{v}(t)-u(x,t)|{}_{x=vt})=0\;$$

(2) $$m^{*}\ddot{u}(x,t)+EIu^{\prime \prime \prime \prime }(x,t)=f_{c}(t)\delta (x-vt)\;$$

where u(x,t) is the vertical displacement of the bridge, $u_{v}(t)$ is the vertical displacement of the vehicle, measured from its static equilibrium position, δ(x−vt) is the Dirac delta function, and the superposed dot and prime denote derivatives with respect to time *t* and coordinate *x*, respectively. The contact force $f_{c}(t)$ is expressed as follows:

(3) $$f_{c}(t)=-m_{v}g+k_{v}(u_{v}(t)-u(x,t)|{}_{x=vt})\;$$

where g is the acceleration of gravity.

Using the modal superposition method, one can obtain the solution for the acceleration response of the test vehicle as follows [18,20]:

(4) $${\ddot{u}}_{v}(t)=\sum_{n=1}^{\infty }\{A_{1,n}\cos\left(\frac{\left(n-1\right)\pi v}{L}\right)t+A_{2,n}\cos\left(\frac{\left(n+1\right)\pi v}{L}\right)t+A_{3,n}\cos\left(\omega_{v}t\right)\;+A_{4,n}\cos\left(\omega_{b,n}-\frac{n\pi v}{L}\right)t+A_{5,n}\cos\left(\omega_{b,n}+\frac{n\pi v}{L}\right)t\}\;$$

where *n* is the counter for the bridge mode, nπv/L is the driving frequency, $\omega_{v}$ is the vehicle frequency, as shown in Equation (5), and $\omega_{b,n}$ is the bridge *n*-th mode frequency identified by FFT, as shown in Equation (6):

(5) $$\omega_{v}=\sqrt{k_{v}/m_{v}}\;$$

(6) $$\omega_{b,n}=\frac{n^{2}\pi^{2}}{L^{2}}\sqrt{\frac{EI}{m^{*}}}\;$$

The coefficients in Equation (4) are given as follows:

(7) $$A_{1,n}=-{\left(\frac{\left(n-1\right)\pi v}{L}\right)}^{2}\times \frac{\Delta_{st,n}\omega_{v}^{2}}{2(1-S_{n}^{2})(\omega_{v}+\frac{(n-1)\pi v}{L})(\omega_{v}-\frac{(n-1)\pi v}{L})}\;$$

(8) $$A_{2,n}=-{\left(\frac{(n+1)\pi v}{L}\right)}^{2}\times \frac{-\Delta_{st,n}\omega_{v}^{2}}{2(1-S_{n}^{2})(\omega_{v}+\frac{(n-1)\pi v}{L})(\omega_{v}-\frac{(n-1)\pi v}{L})}\;$$

(9) $$A_{3,n}=-\omega_{v}^{2}\times \{\frac{2\Delta_{st,n}\omega_{v}^{2}{\left(\frac{\pi v}{L}\right)}^{2}n}{2(1-S_{n}^{2})(\omega_{v}+\frac{(n-1)\pi v}{L})(\omega_{v}-\frac{(n-1)\pi v}{L})(\omega_{v}+\frac{(n+1)\pi v}{L})(\omega_{v}-\frac{(n+1)\pi v}{L})}\;-\frac{2\Delta_{st,n}S_{n}\omega_{v}^{2}\left(\frac{n\pi v}{L}\right)\omega_{b,n}}{\left(\omega_{v}-\omega_{b,n}+\frac{n\pi v}{L})(\omega_{v}+\omega_{b,n}-\frac{n\pi v}{L})(\omega_{v}+\omega_{b,n}+\frac{n\pi v}{L})(\omega_{v}-\omega_{b,n}-\frac{n\pi v}{L}\right)}\}\;$$

(10) $$A_{4,n}={\left(\omega_{b,n}-\frac{n\pi v}{L}\right)}^{2}\times \frac{-S_{n}\Delta_{st,n}\omega_{v}^{2}}{2(1-S_{n}^{2})(\omega_{v}-\omega_{b,n}+\frac{n\pi v}{L})(\omega_{v}+\omega_{b,n}-\frac{n\pi v}{L})}\;$$

(11) $$A_{5,n}=-{\left(\omega_{b,n}+\frac{n\pi v}{L}\right)}^{2}\times \frac{S_{n}\Delta_{st,n}\omega_{v}^{2}}{2(1-S_{n}^{2})(\omega_{v}+\omega_{b,n}+\frac{n\pi v}{L})(\omega_{v}-\omega_{b,n}-\frac{n\pi v}{L})}\;$$

where the vehicle-induced static deflection $\Delta_{st,n}$ of the bridge and the speed parameter $S_{n}$ of the *n*-th mode of the bridge are defined as follows:

(12) $$\Delta_{st,n}=\frac{-2m_{v}gL^{3}}{n^{4}\pi^{4}EI}\;$$

(13) $$S_{n}=\frac{n\pi v}{L\omega_{b,n}}\;$$

To extract the mode shapes of the bridge [25], the component response corresponding to the bridge frequency of the *n*-th mode should be singled out from the vehicle response by a feasible filtering technique based on Hilbert transform [25]:

(14) $$z(t)=R_{b}\left(t\right)+i{\hat{R}}_{b}\left(t\right)=\left[\frac{\omega_{b,n}^{2}S_{n}\Delta_{st,n}\omega_{v}^{2}}{\left(1-S_{n}^{2}\right)\left(\omega_{v}^{2}-\omega_{b,n}^{2}\right)}|\sin\frac{n\pi vt}{L}\right]e^{i\left(w_{b,n}t-\frac{\pi }{2}\right)}\;$$

(15) $$R_{b}=A_{4,n}\cos\left(\omega_{b,n}-\frac{n\pi v}{L}\right)t+A_{5,n}\cos\left(\omega_{b,n}+\frac{n\pi v}{L}\right)t\;$$

(16) $${\hat{R}}_{b}(t)=H\left[R_{b}(t)\right]=A_{4,n}\sin\left(\omega_{b,n}-\frac{n\pi v}{L}\right)t+A_{5,n}\sin\left(\omega_{b,n}+\frac{n\pi v}{L}\right)t\;$$

where the coefficients $A_{4,n}$ and $A_{5,n}$ are defined in equations (10) and (11), respectively. Equation (14) indicates that, in the dynamic response of the test vehicle during its passage over the bridge, the component response of the *n*-th bridge frequency, $\omega_{b,n}$, oscillates with a varying amplitude, but with a shape identical to the *n*-th mode shape of the bridge in a sinusoidal form. In other words, the bridge component response oscillates within the envelope formed by the mode shape of the bridge, as implied by the instantaneous amplitude of the vehicle response.

With the *n*-th frequency and corresponding mode shape of the bridge made available by the procedure presented above, the bending stiffness of each element of the bridge can be calculated by the improved DSC method [8,9,10]. The improvement to the original DSC technique [3,4] is based on the fundamental mechanics of beams, where the bending stiffness *EI* of each cross-section is equal to the modal bending moment *M* at the same cross-section divided by the corresponding modal curvature, namely:

(17) $$EI=\frac{M}{d^{2}\varphi /dx^{2}}=\frac{M}{\kappa }\;$$

where *x* is the axis of the beam, φ is the mode shape function, and κ is the modal curvature. Equation (17) is valid for each mode of the beam if the effects of damping and shear deformation are ignored. This elementary beam theory can be approximately applied for the damage identification of beam structures, along with the indirect identification technique, as discussed in this paper.

According to the D’Alembert’s principle [26], the cross-section of a beam should be in dynamic equilibrium in the presence of inertia force. With the improved DSC method [8,9,10], the internal force at each cross-section can be calculated for the *n*-th frequency and corresponding mode shape. In this study, the modal curvature of the *n*-th mode shape is calculated using the central difference method [27]. With the modal curvature and internal forces made available for each cross-section, the bending stiffness can be calculated for the *n*-th mode of the beam. Based on this approach, the bending stiffness at each node of the bridge model, e.g., using the finite element method (FEM), can be obtained from the frequency-domain method.

In this study, it is assumed that the bridge under investigation is monitored regularly by the test vehicle termly, and the acceleration response is recorded from the test vehicle during each passage. Such a procedure of comparison is repeated for the bridge throughout its service. The following is a summary of the analysis procedure:

(1) It is assumed that a *previous* monitoring of the bridge of concern has been completed using the procedure stated below, which is regarded as the *undamaged* state.
(2) The acceleration response is recorded for the test vehicle during its passage over the bridge for the *current* monitoring, which is suspected as the *damaged* state.
(3) Identify the *n*-th frequency of vibration of the bridge from the recorded vehicle response in the previous and current runs of monitoring.
(4) Recover the *n*-th mode shape of the bridge from the instantaneous amplitude of the component response corresponding to the *n*-th frequency.
(5) Calculate the stiffness *EI* using the *n*-th frequency and corresponding mode shape for the bridge, based on which the structural damage is detected.
(6) If no damage is detected, then the current monitoring is regarded as the *undamaged* state, and the same procedure of damage detection is repeated for the next monitoring.

**Figure 2:**
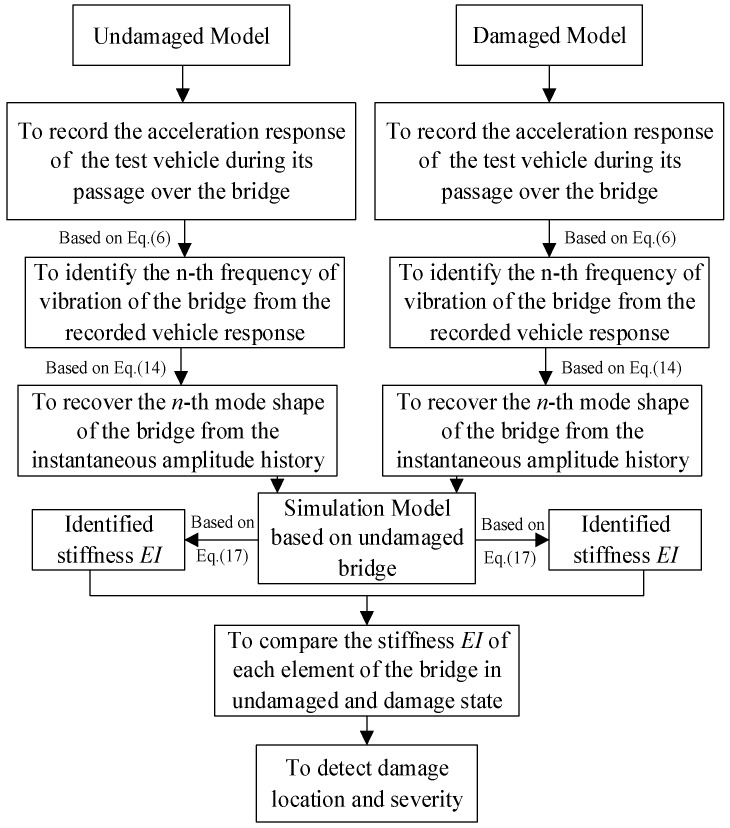
Flowchart of the frequency domain method.

Therefore, the corresponding flowchart is presented in Figure 2.

### 2.2. Time-Domain Method

The statistical moment-based damage detection method is proposed by Xu et al. [5,6] for identifying the stiffness properties of a shear building before and after the occurrence of damages using the measured building story responses. Subsequently, for determining the damage location and severity in the structure, the stiffness properties identified for the two states are compared. It is demonstrated [5,6] that the fourth-order moment, rather than the second-order or the sixth-order moments, of the displacement story response is more suitable for identifying the stiffness properties, as a tradeoff between the sensitivity of the index to structural damage and the stability to random excitation. Such a technique was experimentally verified using shaking table tests for three shear building models [5].

Unlike previous studies [5,6], the fourth-order moment of displacement is adopted herein for the bridge structure using the response data collected by the passing test vehicle. The acceleration response of the test vehicle in Equation (4) can be integrated to yield the displacement response as follows:

(18) $$u_{v}(t)=\sum_{n=1}^{\infty }\{{\bar{\bar{A}}}_{1,n}\cos\left(\frac{\left(n-1\right)\pi v}{L}\right)t+{\bar{\bar{A}}}_{2,n}\cos\left(\frac{\left(n+1\right)\pi v}{L}\right)t+{\bar{\bar{A}}}_{3,n}\cos\left(\omega_{v}t\right)\;+{\bar{\bar{A}}}_{4,n}\cos\left(\omega_{b,n}-\frac{n\pi v}{L}\right)t+{\bar{\bar{A}}}_{5,n}\cos\left(\omega_{b,n}+\frac{n\pi v}{L}\right)t\}\;$$

where the coefficients in the above equation are listed below:

(19) $${\bar{\bar{A}}}_{1,n}=A_{1,n}/-{\left(\frac{(n-1)\pi v}{L}\right)}^{2},{\bar{\bar{A}}}_{2,n}=A_{2,n}/-{\left(\frac{(n+1)\pi v}{L}\right)}^{2},{\bar{\bar{A}}}_{3,n}=A_{3,n}/-\omega_{v}^{2}\;{\bar{\bar{A}}}_{4,n}=A_{4,n}/-{\left(\omega_{b,n}-\frac{n\pi v}{L}\right)}^{2},{\bar{\bar{A}}}_{5,n}=A_{5,n}/-{\left(\omega_{b,n}+\frac{n\pi v}{L}\right)}^{2}\;$$

For the case where the parameters $v,\;L,\;\omega_{v},\;m_{v}$ are constants, the displacement response of the test vehicle is only related to the frequency and bending stiffness of the bridge. In practice, it is assumed that the structural mass remains unchanged before and after damage [3,4,5,6,7,8,9,10]. Thus, the displacement response of the test vehicle is indicative of the bending stiffness of the bridge, which is the property exploited in the following discussion.

In this paper, we assume that a bridge is divided into *N* elements, and that each element has $N_{s}$ sampling points. For the *i*-th element of the bridge, the displacement of the test vehicle can be given as $u_{v_{i}}^{}(i)=[u_{v_{i}}^{}(1),u_{v_{i}}^{}(2),\ldots ,u_{v_{i}}^{}(N_{s})]$ Thus, the average displacement response of the test vehicle at the *i*-th element can be computed as follows:

(20) $${\bar{u}}_{v_{i}}=\frac{1}{N_{s}}\sum_{j=1}^{N_{s}}u_{v_{i}}^{}(j)\;$$

Accordingly, the fourth-order moment vector at each element of the bridge can be computed from the displacement response of the test vehicle as follows:

(21) $${\hat{M}}_{4}=[{\hat{M}}_{41},{\hat{M}}_{42},\ldots ,{\hat{M}}_{4N}]\;$$

where the entry for the *i*-th element is expressed as follows:

(22) $${\hat{M}}_{4i}={\int_{-\infty }^{+\infty }\left(u_{v_{i}}-{\bar{u}}_{v_{i}}\right)}^{4}p\left(u_{v_{i}}\right)du_{v_{i}}\;$$

where $p(u_{v_{i}})$ is the PDF of the structural response $u_{v_{i}}$. Thus, it can be calculated by using summation-type relationships as follows [5,6]:

(23) $${\hat{M}}_{4i}=\frac{1}{N_{s}}\sum_{j=1}^{N_{s}}u_{v_{i}}{\left(j\right)}_{}^{4}-\frac{4}{N_{s}}{\bar{u}}_{v_{i}}\sum_{j=1}^{N_{s}}u_{v_{i}}{\left(j\right)}_{}^{3}+\frac{6}{N_{s}}{\bar{u}}_{{}_{v_{i}}}^{2}\sum_{j=1}^{N_{s}}u_{v_{i}}{\left(j\right)}_{}^{2}-3{\bar{u}}_{{}_{v_{i}}}^{4}\;$$

First, an initial value is assign to the stiffness *EI* of the bridge using the value obtained from the *previous* monitoring. With this value, the vehicle response can be solved from Equations (1) and (2). Then, the fourth-order moment vector corresponding to the *previous* monitoring can be computed from Equations (18), (21), and (23), which is considered as the theoretical statistical moment vector, $M_{4}=[M_{41},M_{42},\ldots ,M_{4N}]$. Simultaneously, the fourth-order moment vector, ${\hat{M}}_{4}$, can be computed using the test vehicle response recorded during the *current* monitoring. Therefore, the residual vector between $M_{4}$ and ${\hat{M}}_{4}$ is calculated as follows:

(24) $$F(EI)=M_{i}(EI)-{\hat{M}}_{i}\;$$

Ideally, if the given vector of the stiffness values of all of the elements *EI* is equal to the actual values, the two norms of the residual vector, ‖F(EI)‖, becomes zero. Practically, the vector of the optimal stiffness values can be identified by the least-squares method. Giving the *EI* of an element an initial value $EI_{0}$ from a *previous* monitoring, compute the corresponding fourth-order member moment, compare the actual value of the fourth-order member moment established from the *current* monitoring to that computed from the initial value $EI_{0}$, and finally, minimize ‖F(EI)‖ to assess the damage condition of the bridge. Based on this approach, the bending stiffness at each element can be obtained.

The time-domain method in the indirect identification technique can be evaluated according to the analysis procedure of the following steps:

(1) Measure the displacement responses of the test vehicle, or calculate the displacement from the acceleration response of the test vehicle during its passage over the bridge for the undamaged and damaged states.
(2) The actual statistical moments of the measured displacement responses of the test vehicle with the undamaged and damaged states, ${\hat{M}}_{4i}$, are estimated using Equation (23).
(3) Given the vector that collects all of the stiffness parameters for all of the elements representing the bridge FE model using initial values based on the calculated stiffness, e.g., from the frequency-domain method, the theoretical statistical moments of the displacement responses of the test vehicle, $M_{4}$, are calculated based on the FE model of the bridge and also making use of Equation (23).
(4) Substituting ${\hat{M}}_{4}$ and $M_{4}$ into Equation (24), the vector collecting the structural stiffness values of all of the elements of the FE model of the bridge can be identified by the constrained nonlinear least-squares method for the undamaged and damaged states.
(5) All of the attributes of the structural damage of the bridge, including the existence, location, and severity, can be detected by comparing the identified vector of the stiffness values of the undamaged bridge, $\hat{E}I^{u}$, to that of the damaged bridge, $\hat{E}I^{d}$.

**Figure 3:**
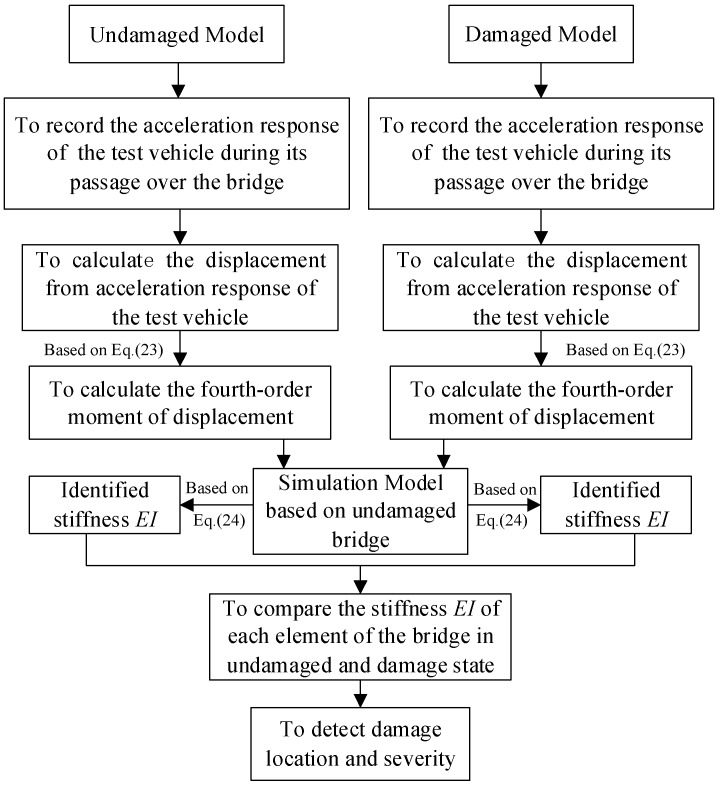
Flowchart of the time-domain method.

Therefore, the corresponding flowchart is presented in Figure 3.

## 3. Parameters of the Test Vehicle Numerical Study

**Figure 4:**
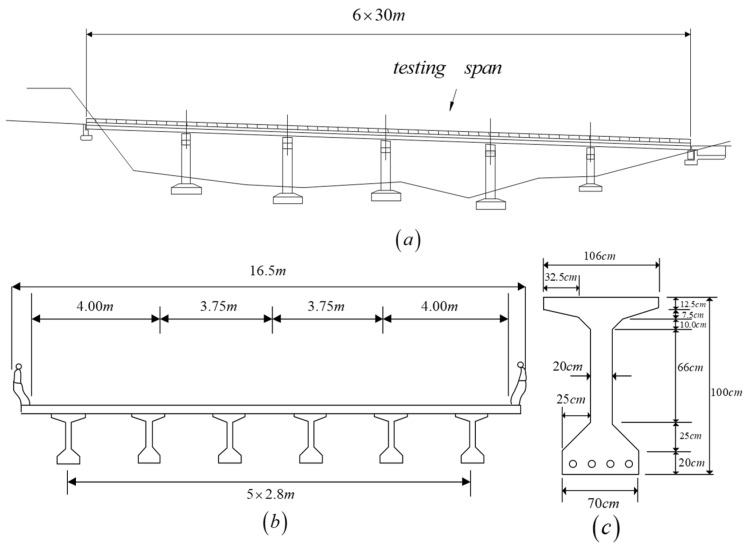
Bridge considered for the simulation, (**a**) bridge elevation, (**b**) bridge cross-section, (**c**) girder cross-section.

**Figure 5:**
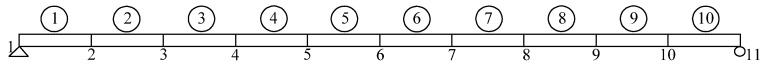
Finite element (FE) model of one span of the bridge with 10 elements.

In order to investigate the feasibility and limitations of the presented approaches from the dynamic response of the passing test vehicle, several numerical cases are studied herein using the FEM, based on a well-developed simulation algorithm for vehicle–bridge interaction [17,25]. For the considered numerical simulation, the simply supported bridge is one span of the Da-Wu-Lun bridge [19], which is a part of the Taiwan Provincial Highway 2 near the northern coast of Taiwan. The considered bridge unit is composed of six prestressed I girders, placed at a center-to-center distance of 2.8 m, and has a span length of 30 m, as shown in Figure 4. The cross-section of the bridge has a total width of 16.5 m with a 20-cm thick concrete deck slab and a five-cm thick Asphalt Concrete(AC) pavement layer. The cross-sectional area and moment of inertia of each I girder are 0.64 m^2^ and 0.2422 m^4^, respectively. The concrete of the bridge has an elastic modulus of 29 GPa and a material density of 2400 kg/m^3^. Figure 5 shows the FE model of the considered bridge span with 10 beam elements (i.e., 11 nodes) where the numbers in circles are the element numbers, and the others are the node numbers.

**Figure 6:**
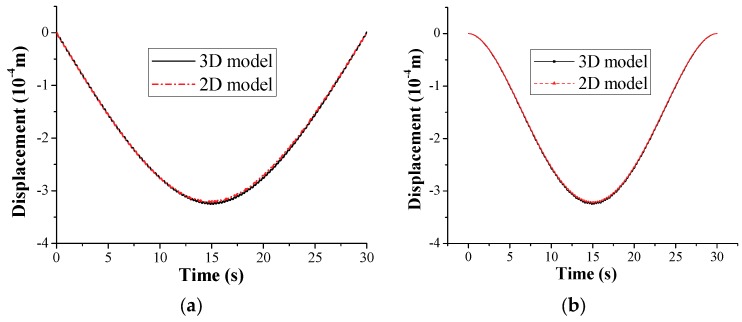
Vertical displacement response of (**a**) bridge midpoint and (**b**) test vehicle.

The accuracy of the single-mode closed-form solution obtained for the vehicle–bridge couple system, and the vehicle response in particular, will be verified by the three-dimensional elements and two-dimensional elements for a typical example. As for the above bridge modal, the following data are adopted for the test vehicle: mass *m_v_* = 500 kg, stiffness *k_v_* = 90 kg/m, *v* = 1 m/s, and zero damping. For this vehicle, the vehicle to bridge mass ratio is 1:100. The vertical displacement of the vehicle obtained by the three-dimensional element and two-dimensional element approaches have been plotted in Figure 6a,b, respectively. As can be seen from Figure 6 and all of the analyzed results, the solutions obtained by the two approaches show a high degree of coincidence for the vehicle response; however, the analytical results are considered acceptable for the purpose of identifying the key parameters involved. Therefore, studying the frequency-domain method and time-domain method of the indirect measurement technique as the key point, the simulation model below are all based on the two-dimensional element approach for simplicity.

**Figure 7:**
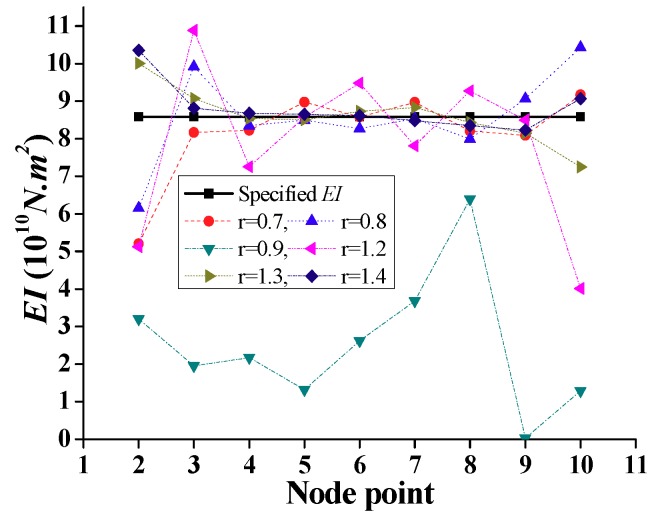
Simulation results of the calculated *EI* for elements along the bridge for different *r* values.

With the test vehicle acceleration and displacement responses discussed above, several test vehicle parameters are required for the indirect technique of bridge damage identification. The test vehicle parameters are frequency $\omega_{v}$, mass $m_{v}$, stiffness $k_{v}$, and speed *v*. Considering the frequency-domain method and based on previous studies [17,25], the ratio of the bridge fundamental frequency $\omega_{b}$ to $\omega_{v}$, i.e., $r=\omega_{b}/\omega_{v}$, is an important design parameter of the field test for the intended purpose of stiffness identification. In this study, a test vehicle with $m_{v}=500$ kg and *v* = 3 m/s, $k_{v}$ is adjusted for the different values of *r*, and the *EI* of the bridge is computed. Figure 7 shows results corresponding to *r* = 0.7 to 1.4 (for nodal point numbers, refer to Figure 5). It is noted that the nodes located in the neighborhood of the abutments (nodes 2 and 10) did not correspond to accurate results because of the unsuitable combination of the identified mode shape from the test vehicle [25] and the improved DSC method [8,9,10] in the frequency-domain method. This is attributed to the higher errors of the identified mode shapes near the boundaries compared to near the mid-span. Except for these nodes, it is shown that when *r* = 0.7 and 1.4, the calculated *EI* is closed to the specified *EI* where the difference is below 5%. With *r* approaching 1.0 from above or below, the calculated *EI* becomes coarser due to resonance where the bridge vibration includes significant vehicle vibration in the same frequency band. Accordingly, the calculated *EI* is inaccurate compared with the specified *EI*; refer to the result for *r* = 0.9 in Figure 7. An important point follows from this discussion, namely, if the natural frequency of the test vehicle is close to the natural frequency of the bridge, it is difficult to use the frequency-domain method for damage identification, because the collected data include a similar frequency signal for the test vehicle and the bridge. Therefore, for the ratio *r* ≤ 0.7 or *r* ≥ 1.4, the identification of *EI* is suitable for damage identification.

**Figure 8:**
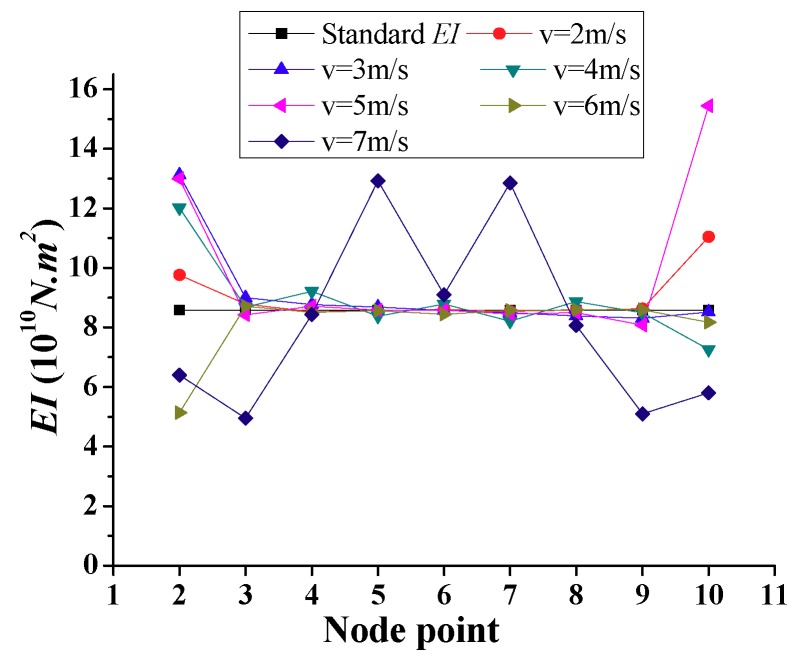
Simulation results of calculated *EI* with different test vehicle speeds.

Another considered factor is the constant speed of the test vehicle, v. The calculated *EI* using the frequency-domain method is shown in Figure 8 for speeds ranging from 2 m/s to 7 m/s. It is shown that the calculated *EI* should be based on the vehicle speed not exceeding 6 m/s to obtain suitable stiffness identification, not including boundary elements, i.e., nodes 2 and 10.

## 4. Considered Scenarios in the Numerical Study

Based on the previous section, the following values are considered for the main parameters of the test vehicle in this section: mass *m_v_* = 500 kg, stiffness *k_v_* = 90 kg/m, and *v* = 3 m/s. The finite element (FE) model of the bridge in Figure 5 is adopted with a time step of 0.01 s to demonstrate the sensitivity of the detection methods. The acceleration and displacement responses that are numerically generated for the test vehicle during its passage over the bridge are processed using the procedures outlined above to identify the bridge bending stiffness (*EI*). The considered damage scenarios are listed in Table 1 and identified by D, with a subscript indicating the damaged element number. The severity of the damage is denoted by the percentage of reduction from the original (undamaged) sectional bending stiffness.

### 4.1. Frequency-Domain Method

The fundamental frequency and corresponding mode shape are accurate and convenient regarding extraction from the acceleration responses of the test vehicle [25]. When the improved DSC method [8,9,10] is used, only the measurements in one mode are sufficient to identify the damage. Using the indirect identification technique, only the fundamental frequency and the corresponding mode shape are used to calculate the bending stiffness at the nodes herein.

**Figure 9:**
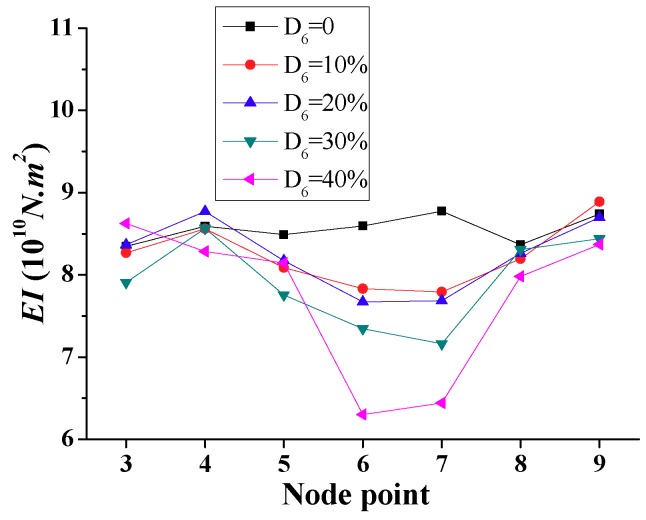
Simulation results of calculated *EI* for a single damage (Scenario 1).

**Figure 10:**
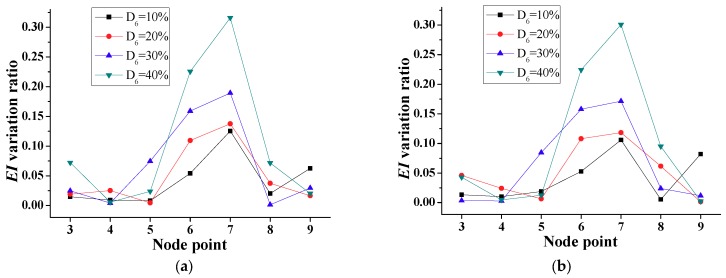
*EI* variation ratios for a single damage (Scenario 1). (**a**) Ratio with respect to calculated undamaged *EI*. (**b**) Ratio with respect to specified undamaged *EI*.

To demonstrate the damage detection for a single damage location using the frequency-domain method, it is assumed that the bridge in Figure 9 experienced damage Scenario 1 (Table 1). From Figure 9, it is clear that the stiffness values at nodes 6 and 7, corresponding to D_6_, are the lowest. Figure 10 shows the *EI* variation ratio, i.e., stiffness degradation level, at the different nodes. For the undamaged state, the corresponding stiffness values at nodes can be calculated from the indirect identification technique of from the original design documents. Figure 10a,b shows the variation ratio considering the undamaged *EI* similar to the on-site test immediately after a newly constructed bridge using the indirect identification technique, and the specified undamaged *EI* similar to the original design document, respectively. It is indicated that the *EI* variation ratios at nodes 6 and 7 are close to the true values, i.e., the mean values of the damage percentages of the adjacent elements (D_6_ + D_5_)/2 for node 6 and (D_6_ + D_7_)/2 for node 7. Thus, the distribution of *EI* and the corresponding variation ratio along the bridge are satisfactory for detecting the damage location and severity, except in boundary elements 1 and 10.

**Figure 11:**
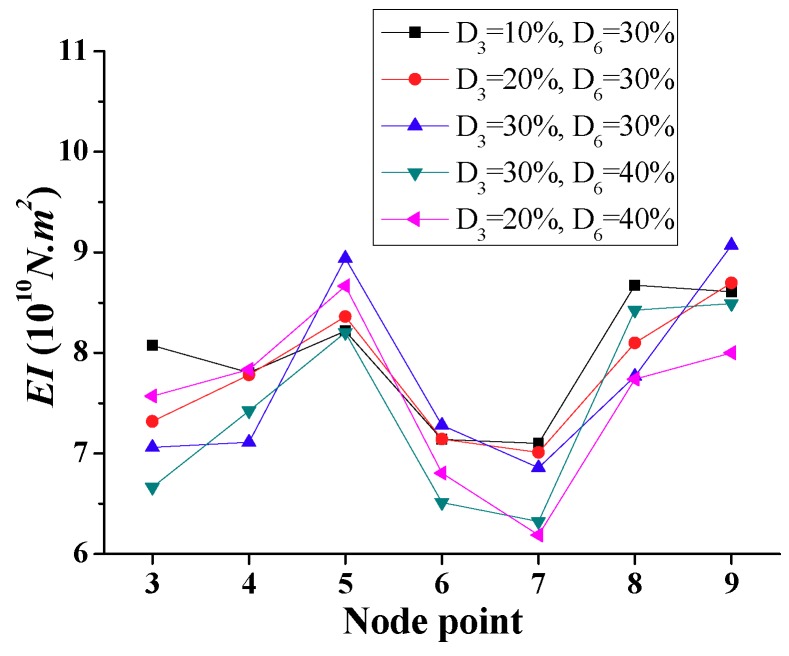
Simulation results of calculated *EI* for double interior damages (Scenario 2).

**Figure 12:**
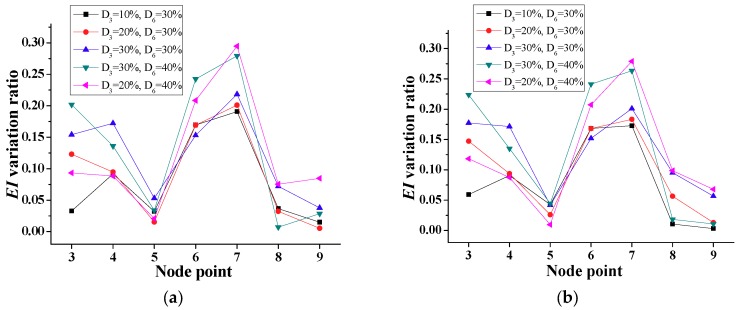
*EI* variation ratio for double interior damages (Scenario 2). (**a**) Ratio of calculated to undamaged *EI*. (**b**) Ratio of specified to undamaged *EI*.

Figure 11 shows the calculated *EI* in the case of multiple damage locations (Scenario 2 of Table 1) corresponding to the damaged element 3 (nodes 3 and 4) and element 6 (nodes 6 and 7). Moreover, Figure 12a,b indicates the variation ratio with respect to the calculated and specified undamaged *EI*, respectively. The simulation results indicate that the magnitudes of the variation ratio increase with the increase of damage severity.

Similar observations can be made for scenarios 1 and 2, as shown in Table 1. Element 6 is damaged in both scenarios, with only one damaged element in Scenario 1, and two damaged elements in Scenario 2. The calculated *EI* and corresponding variation ratio exhibit almost no change for element 6 in these two scenarios with the same assumed damaged case. This important feature of the ability to detect damage locations and severity without the influence of damage of other locations is essential for practical applications of structural damage identification.

### 4.2. Time-Domain Method

**Figure 13:**
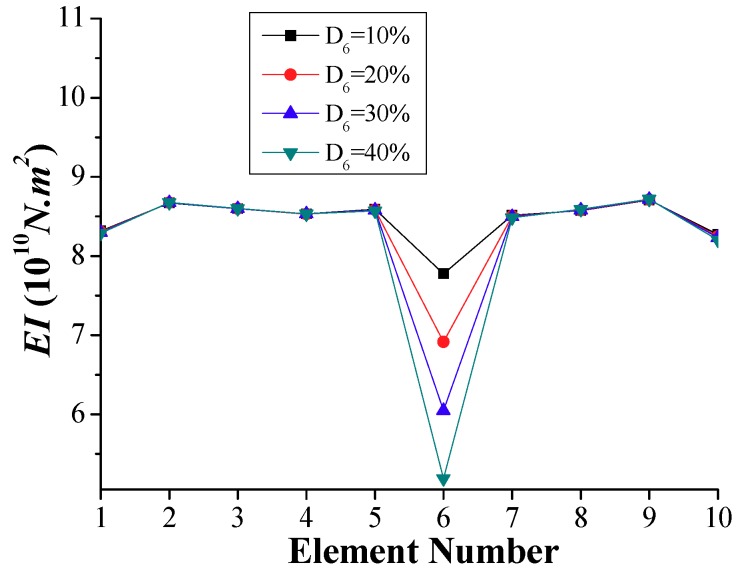
Simulation results of calculated *EI* for a single damage (Scenario 1).

**Figure 14:**
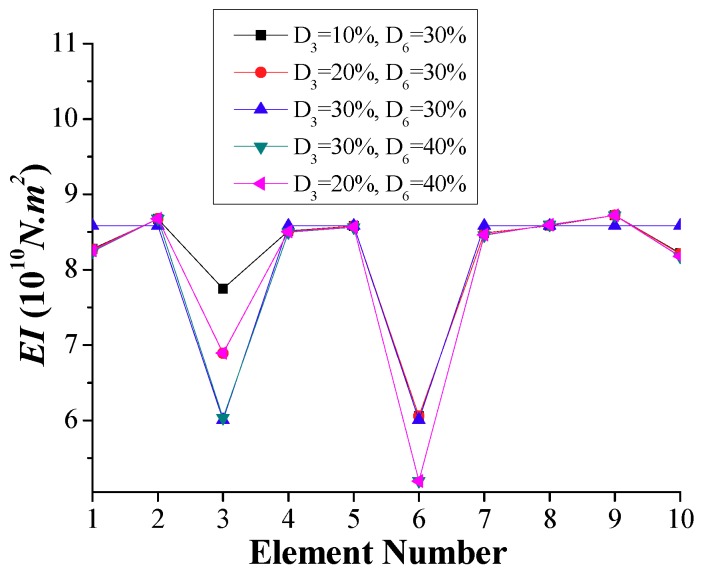
Simulation results of calculated *EI* for two interior damages (Scenario 2).

**Figure 15:**
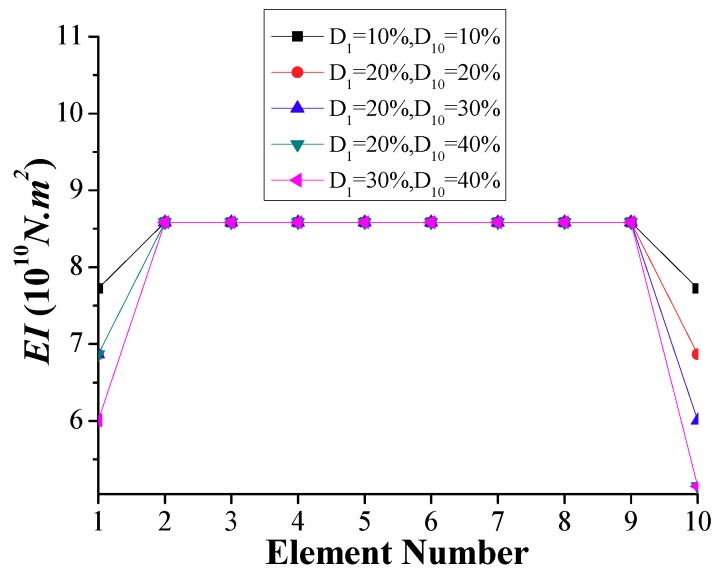
Simulation results of calculated *EI* for boundary damages (Scenario 3).

Using Equation (21), the fourth-order moment vectors of the “measured” displacement response of the test vehicle can be estimated for the previously discussed bridge model and test vehicle parameters considering different damage scenarios. Thus, the consequent *EI* of each element, which may differ from the calculated *EI* at each node using the frequency-domain method, can be calculated. The identified *EI* of each element is represented in scenarios, as shown in Figure 13, Figure 14 and Figure 15.

According to the time-domain method, the distribution of stiffness is determined for each element as shown in Figure 13, showing the lowest stiffness for the damaged element 6. The computed ratios of reduction in stiffness for this element of 0.093, 0.194, 0.295, and 0.396 are very close to the damaged cases, i.e., D_6_ = 10%, 20%, 30%, and 40%, respectively. On the other hand, the variations of stiffness in undamaged elements of Figure 13 are very small, below 5%.

When two damaged inner elements, 3 and 6, are simulated (Scenario 2), the identified stiffness at each element, as shown in Figure 14, is accurate compared to the specified distribution of stiffness. The differences of the identified stiffness values and those of the specified ones for the damaged and undamaged elements are below 1% and 5%, respectively. As stated previously, the boundary elements cannot be identified properly using the frequency-domain method. However, the time-domain method permits the *EI* of the boundary elements to be accurately calculated, as shown in Figure 15 for the damaged stiffness of boundary elements 1 and 10. The variations of the stiffness between the identified stiffness values and the specified ones are below 1%.

Based on the above results, the time-domain method can be used for boundary damage identification where the frequency-domain method cannot. However, the frequency-domain method is more efficiently computationally compared to the time-domain method, which requires solving an optimization problem. For the discussed bridge simulation, calculating *EI* using the time-domain method starting with specified undamaged stiffness for the initial values at each element may require extensive computations. However, if the initial stiffness values are identified from the application of the frequency-domain method, the computing time that is required to solve the optimization problem by applying the time-domain method can be significantly reduced.

## 5. Measurement Error Study

For a reliable damage detection method, a significant challenge is posed by environmental noise in practical applications, e.g., thermal conditions or the effects of the roughness of the road surface. In this study, it is assumed that the influence of the environmental effects on the dynamic response of the passing test vehicle is represented by *white noise*. The displacement response of the test vehicle with random noise is expressed as follows [28,29]:

(25) $$y_{m}=y_{calculated}+E_{p}P\sigma \left(y_{calculated}\right)\;$$

where $y_{calculated}$ is the calculated displacement and acceleration responses of the test vehicle from the FE model, $E_{p}$ is the noise level, *P* is an independent random variable of Gaussian distribution with zero mean and unit standard deviation, and $\sigma \left(y_{calculated}\right)$ is the specified standard deviations of the calculated displacement responses of the test vehicle.

### 5.1. Considering Noise in the Frequency-Domain Method

**Figure 16:**
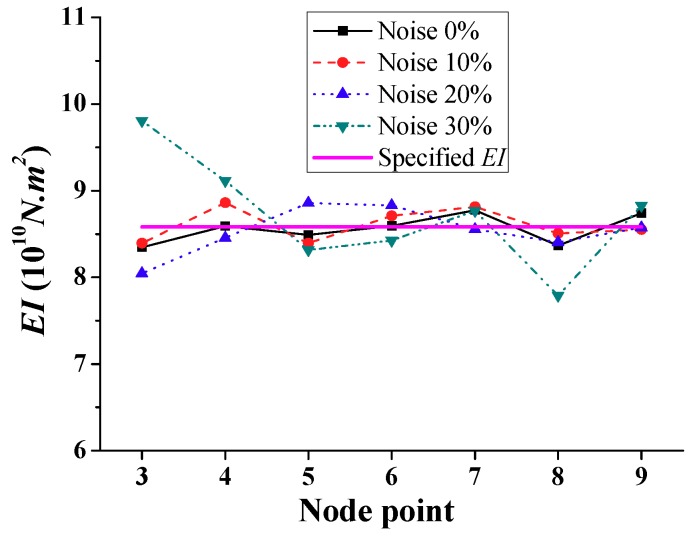
Simulation results of calculated *EI* with different noise levels.

**Figure 17:**
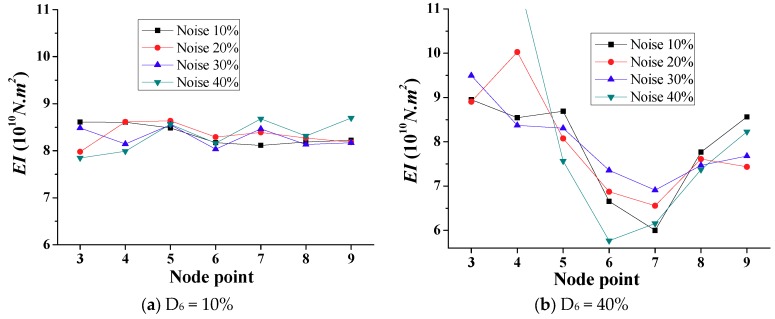
Simulation results of calculated *EI* with different noise levels (Scenario 1).

**Figure 18:**
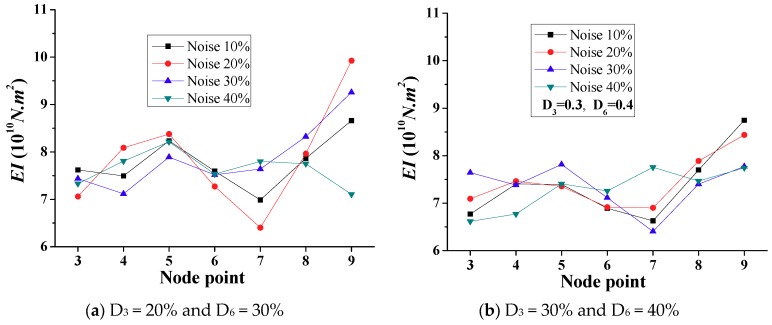
Simulation results of calculated *EI* with different noise levels (Scenario 2).

To numerically demonstrate the sensitivity of the identified bending stiffness using the frequency-domain method, it is assumed that the discussed bridge simulation experienced the scenarios that are summarized in Table 1, but with a consideration of different noise levels, as shown in Figure 16, Figure 17 and Figure 18. Using Equation (25), the simulations with added Gaussian random white noise for each level of the bridge are repeated 10 times in order to reduce the effects of the random errors (similar to 10 *in situ* measurements). The average of the noisy data is used for the subsequent damage detection to estimate the bending stiffness at nodes by the frequency-domain method. Figure 16 shows the identified stiffness values of the undamaged case from the calculated results of the 10 random realizations corresponding to each considered noise level. As expected, the bending stiffness identified from the first mode shape with comparatively low noise level is more accurate than that with higher noise level. This indicates that the variation ratios between the identified and the specified *EI* values are below 3%, even if noisy data are used with up to a 20% noise level. Moreover, the stiffness can be reasonably identified, even for up to 30% noise levels, except for node 3, where the errors are over 20%.

To evaluate the proposed approach for calculating the bending stiffness under different damage scenarios (similar to the above case of the undamaged bridge) with environmental noise, the numerical simulations considering the artificial noise prescribed with different levels are performed under these damaged scenarios. Figure 17 shows the calculated *EI* from the data with the noise at different levels for damaged element 6 with a 10% and 40% reduction in stiffness. From Figure 17a, the results of the damage identification at a 10% noise level are quite satisfactory, and the lowest *EI* values are observed at nodes 6 and 7 (the end nodes of damaged element 6), identifying the damaged location accurately. At the higher noise levels, the proposed approach also revealed the stiffness reduction at node 6. However, the *EI* values at the boundary nodes are even lower than those in the specified damaged regions at the noise level of 30%, which would pose an inevitable impediment for the efficient damage identification. Furthermore, when the bending stiffness of element 6 is presumed to have a 40% reduction in stiffness, all of the results at different noise levels meet the requirements of determining the damage location based on the lowest identified *EI* values, as shown in Figure 17b, where the lower *EI* values at nodes 6 and 7 are apparent. Moreover, as expected, due to the increased noise, larger random errors in the calculated *EI* are observed compared with the specified *EI*. For the scenario of high environmental noise, the curvature that fluctuates κ in Equation 17 would fluctuate much more due to the amplification of differential effects with the $d^{2}\varphi /dx^{2}$ [8,9,10]. Accordingly, the calculated *EI* based on the classical beam theory, Equation (17), may lead to an unreasonable stiffness value, especially in the vicinity of damaged element(s) with the highest chance of a much higher curvature variation [8,9,10], and near support element(s) with the near-zero curvature [8,9,10]. Due to the limited number of repeated numerical simulations, the results are non-ergodic [30], and the averaging techniques can hardly eliminate the interference effects of the Gaussian random noise. Therefore, the “real” signal becomes largely contaminated, resulting in increased or decreased values of the measured data at some positions. Fortunately, these results indicate that the environmental noise would exert smaller influences on the damage identification results when the bending stiffness of the damaged element is significantly reduced.

Figure 18 shows the calculated *EI* with different noise levels for double-interior damages (D_3_ = 20% and D_6_ = 30% versus D_3_ = 30% and D_6_ = 40%). The reduced stiffness of the two damaged elements are generally identified for different noise levels. In addition, the results demonstrate the higher damage of element 6 for most of the noise scenarios. However, the calculated results for the 40% noise level that is shown in Figure 18b illustrate the lower *EI* values at nodes 3 and 4 compared with those at nodes 6 and 7. Therefore, the high environment noise levels would deteriorate the efficiency and accuracy of the proposed frequency domain approach to some extent, and make the damage identification more complex and less reliable.

### 5.2. Considering Noise in the Time-Domain Method

**Figure 19:**
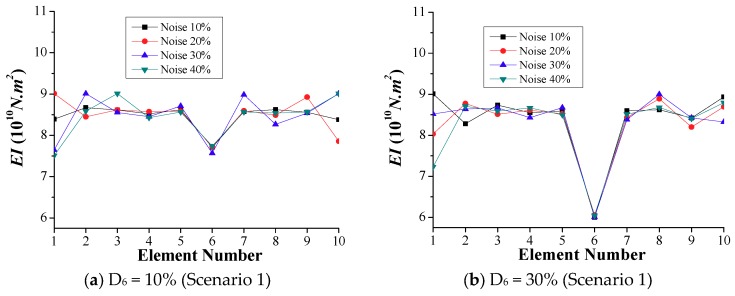

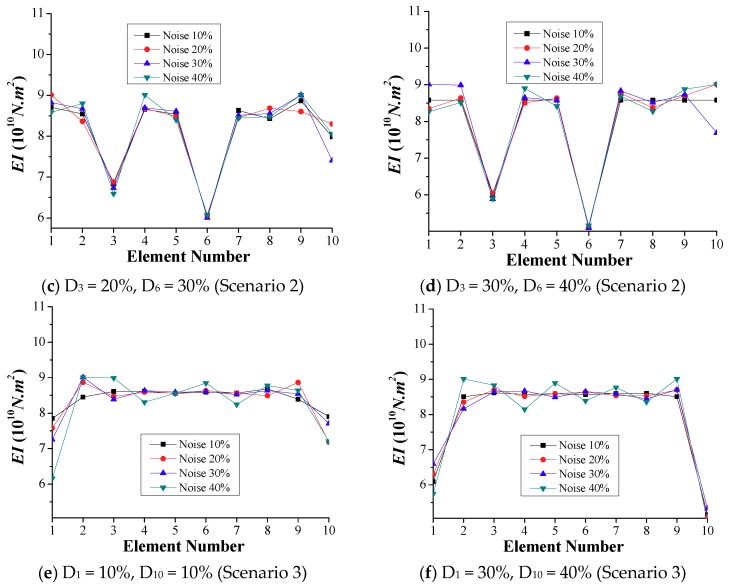
Simulation results of calculated *EI* with different noise levels.

This section presents the effects of the environmental noise on damage identification using the time-domain method. Besides the single and double-element damage scenarios, the scenario of damaged boundary elements is also included. As discussed in the previous section, the calculation following Equation (25) is repeated 10 times, and the averaged results are attained for the analysis. Figure 19 shows the calculated *EI* at different noise levels for the above-mentioned damage scenarios using the time-domain method. It is observed that the differences between the *EI* values of the damaged element(s) are negligible for the different noise levels. Therefore, the environmental noise has insignificant effects on the calculated stiffness when using the time-domain method. In addition, from Figure 19a,b, the damage of element 6 is clear, and the higher reduction of the bending stiffness is accurately reproduced. However, the undamaged boundary elements 1 and 10 can be mistaken for “damaged” elements due to the calculated stiffness reduction at high noise levels. This may lead to unnecessary inspection fieldwork in the case of slightly damaged elements, as shown in Figure 19a. For the scenario of double-element damage, Figure 19c, d reveals the accurate damage locations and damage severity. The effects of the environmental noise and the interference between the two damaged elements are negligible. Unlike the frequency-domain approach, the damages at the boundary elements are well recognized by the time-domain method, as shown in Figure 19e,f. Consequently, the time-domain method is advantageous, with higher accuracy, robustness, and reliability than the frequency-domain method.

## 6. Field Test Study

**Figure 20:**
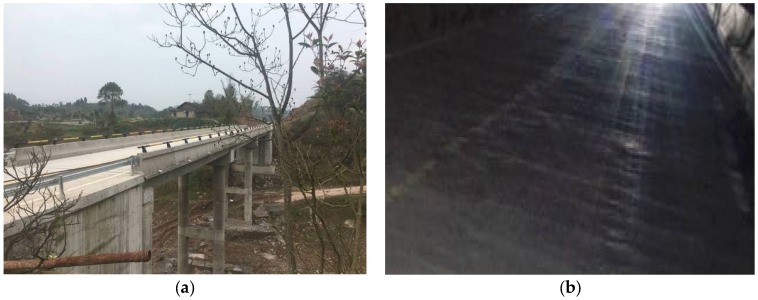
Hong Xing Bridge of Chongqing City.

The Hongxing bridge, located in Fuling District of Chongqing City, is a simply supported three-spanned bridge with each span’s length at 20 m, as shown in Figure 20a. The cross-sectional moment of inertia is 0.38 m^4^, and the elastic modulus is 3.0 × 10^10^ N/m^2^. The bridge was recently built in 2018, and has not been officially open to the public. Therefore, it had little traffic flow, and noise interference was relatively weak. According to field investigations, the road roughness is shown in Figure 20b; it is suitable for the actual experimental study of the indirect measurement technique.

**Figure 21:**
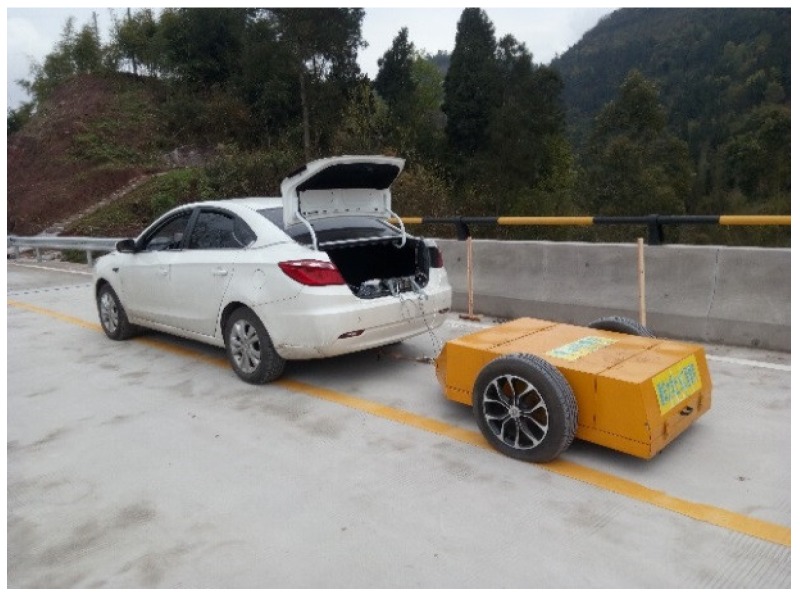
Field tests on site.

**Figure 22:**
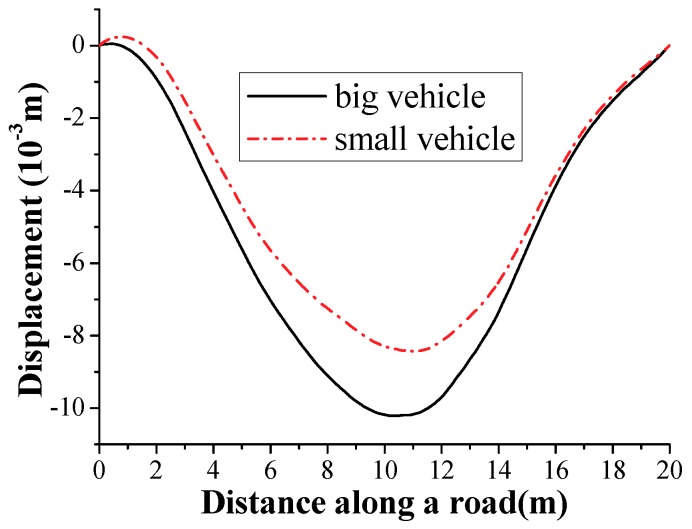
The average of three displacement responses on site.

Considering the second span as the test beam bridge, researchers kept the speed of the test vehicle–car (tractor) system at 1 m/s, as shown in Figure 21. According to the test vehicle going across the test beam bridge, the acceleration response of the test vehicle with an acceleration sensor installed in the center of the test vehicle could be recorded. For reducing the effect of the surface road surface, two different weights of the test vehicle, namely a big vehicle (1100 kg) and small vehicle (1050 kg) with the same vehicle frequency, could pass the test beam bridge, respectively. The difference between the responses of the two test vehicles could be regarded as the initial acceleration response signals. Researchers let the big vehicle and small vehicle pass the second span of the bridge three times, respectively, and then were able to use displacement response-measuring technology based on the double integral of the recorded acceleration response with zero initial conditions at each time. Therefore, there are three displacement responses each for the big vehicle and small vehicle. After averaging the displacement responses of the big vehicle and the small vehicle for reducing random noise, which is shown in Figure 22, the difference between the displacement response of the big vehicle and the small vehicle can be regarded as the initial displacement response signal for the analysis procedure of the time-domain method in Section 2.2. The corresponding acceleration response calculated by the initial displacement response differential twice can be regarded as the initial acceleration signals for an analysis procedure of the frequency-domain method in Section 2.1.

**Figure 23:**
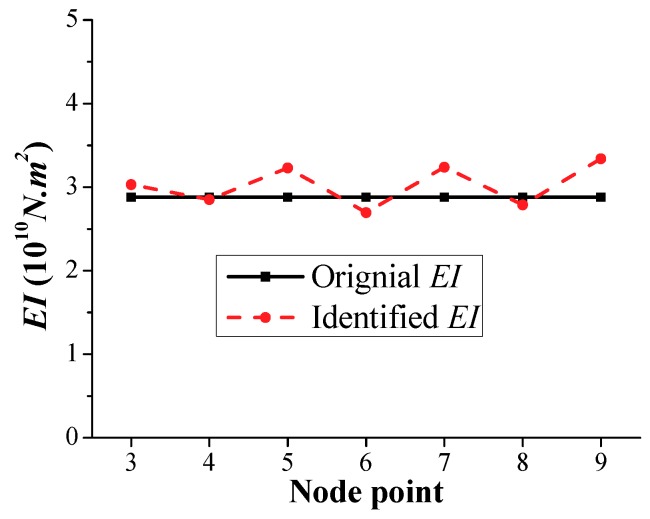
Identified *EI* results calculated by the frequency-domain method.

Based on the analysis procedure of the frequency-domain method in Section 2.1 and time-domain method in Section 2.2, considering the same element and node numbers shown in Figure 5, the identified stiffness *EI* at the element nodal points calculated by the frequency-domain method is shown in Figure 23. Compared to the original *EI*, the maximum relative error in the identified stiffness *EI* occurs in the element node point number 9 with a value of approximately 16%; however, the identified results are acceptable within an engineering acceptance range.

**Figure 24:**
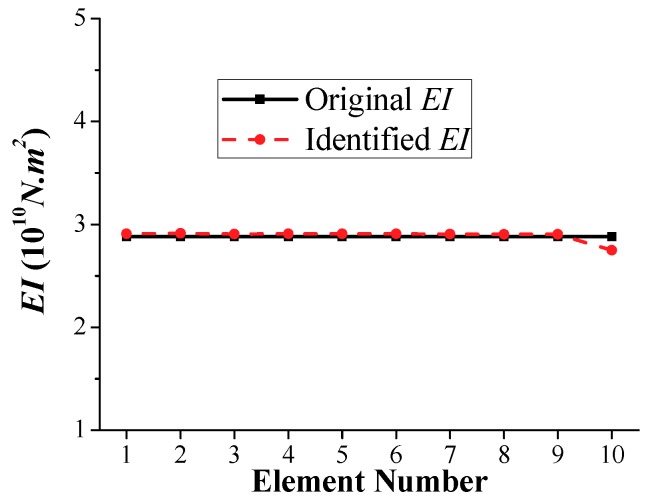
Identified *EI* results calculated by time-domain method.

It can also be seen that the identified stiffness *EI* at the element number calculated by the time-domain method is shown in Figure 24, which is obviously better than the results of Figure 23. Compared with the original *EI*, the maximum relative error in the identified stiffness *EI* occurs in element number 10 with a value of approximately 5%, and the remaining *EI* results are all below 1%. It indicated again that the time-domain method is advantageous with higher accuracy, robustness, and reliability than the frequency domain method.

This is a preliminary verification for an indirect measurement technique. It is noted that the applicability of the frequency-domain method and time-domain method to practical bridges with recorded data from field tests should be further promoted, and details will be presented in future publications.

## 7. Results Discussion

In the simulation numerical, the results indicated the ability to detect damage locations and severity without the influence of damage at other locations in single and double-damage location(s). Based on the sensitivity of different environment noise levels to damage identification, it is noted that higher environment noise would deteriorate the efficiency and accuracy of the proposed frequency-domain approach to some extent, and make the damage identification more complex and less reliable. However, the time-domain method is advantageous with higher accuracy, robustness, and reliability than the frequency-domain method. A filtering method to reduce the measurement noise should be studied in the future.

In the field test, performing multiple passes on the bridge and then averaging the signals was considered to reduce the measurement noise. Two different weight test vehicles with the same vehicle frequency were used for reducing the effect of road surface roughness. The results show that the identification can be accepted as an engineering requirement. More technologies and an updated designed test vehicle to eliminate these disturbance factors will be promoted in future work.

## 8. Conclusions

This study presents an indirect approach for identifying the structural damage of a bridge from a passing test vehicle. Both the frequency-domain and time-domain methods have been embedded into the proposed indirect approach, which is numerically examined in the single, double, and boundary-damage scenarios considering different noise levels. During the passage of the test vehicle over a bridge, the fundamental frequency and corresponding mode shape of the bridge can be extracted from the field measurements recorded by the vibration sensors mounted on the test vehicle. Subsequently, the stiffness for structural damage identification can be calculated from the improved direct stiffness calculation technique, which is referred to as the frequency-domain method. For the displacement response measured by the test vehicle, or the twice integration of the acceleration response, the fourth-order moment vectors can be calculated from the statistical moment-based damage detection method combined into the indirect approach, which is referred to as the time-domain method. Through a numerical case study, the main conclusions are as follows:

1. The proposed indirect approach, including the frequency-domain and time-domain methods, requires no parametric inputs, which is more general compared to other structural damage identification, e.g., wavelet-based methods. Therefore, the proposed approach can be directly adopted for the structural damage identification of in-service bridge structures without additional and cumbersome calibration.
2. The frequency-domain method is advantageous with its high cost efficiency, since it can estimate the initial stiffness of the bridge based on the first mode of vibration, and is sufficient for identifying damage location(s) apart from the end regions of the bridge. However, this method requires that the speed of the passing vehicle should be lower than 6 m/s during the measurement, and it is not applicable for damage identification in the boundary nodes.
3. Although the time-domain method is computationally intensive due to the additional optimization steps, it has the advantages of high accuracy, reliability, and robustness, and is feasible for use especially in the end regions of the bridge, which is suitable for identifying damage location(s) and damage severities.
4. The field test study shows that the identified results errors from using the frequency-domain method and time-domain method are below 16% and 5% respectively; this indicates that the two methods are useful for assessment bending stiffness with a practical bridge. Moreover, it indicated that the time-domain method is advantageous with higher accuracy, robustness, and reliability as compared to the frequency-domain method.
5. In the practical assessment of the bridge health conditions, the frequency-domain approach is suitable in the preliminary phase to estimate the initial damage conditions of the bridge on site. Subsequently, in the final phase of the investigation, the time-domain approach can provide more detailed and comprehensive results with high accuracy and reliability.

Since the conclusions are drawn from the analytical analysis, numerical simulations, and initial field-test verification, as in the practical applications of the proposed damage detection approach for damage identification based on old damaged bridge test, are not included in this paper, and will be presented in future publications. It is noted that the modified bending stiffness results by using the frequency-domain method, especially for the end regions of the bridge, and high measurement noise should be further promoted, and will be presented in future publications. Finally, future research should focus on developing techniques and equipment for designing a test vehicle and considering it without road closure, and more corresponding parameters with a vehicle–bridge couple system associated with practical challenges should be studied.

## Figures and Tables

**Table 1:** Description of damage scenarios in the numerical simulations.

Scenario	Group	Damaged Element(s)	Reduction in Element Stiffness (%)				
1	D_6_	6	D_6_ = 0	D_6_ = 10	D_6_ = 20	D_6_ = 30	D_6_ = 40
2	D_3_, D_6_	3 & 6	D_3_ = 10 D_6_ = 30	D_3_ = 20 D_6_ = 30	D_3_ = 30 D_6_ = 30	D_3_ = 30 D_6_ = 40	D_3_ = 20 D_6_ = 40
3	D_1_, D_10_	1 & 10	D_1_ = 10 D_10_ = 10	D_3_ = 20 D_6_ = 20	D_3_ = 20 D_6_ = 30	D_3_ = 20 D_6_ = 40	D_3_ = 30 D_6_ = 40
